# The Collection of Zoosporic Eufungi at the University of Michigan (CZEUM): introducing a new repository of barcoded *Chytridiomyceta* and *Blastocladiomycota* cultures

**DOI:** 10.1186/s43008-020-00041-z

**Published:** 2020-10-06

**Authors:** D. Rabern Simmons, Anne E. Bonds, Buck T. Castillo, Rebecca A. Clemons, Alex D. Glasco, Jillian M. Myers, Natasha Thapa, Peter M. Letcher, Martha J. Powell, Joyce E. Longcore, Timothy Y. James

**Affiliations:** 1 Department of Ecology and Evolutionary Biology, University of Michigan, Ann Arbor, MI 48109 USA; 2 Department of Biological Sciences, The University of Alabama, Tuscaloosa, AL 35487 USA; 3 School of Biology & Ecology, University of Maine, Orono, ME 04469 USA

**Keywords:** Cryopreservation, Oxford Nanopore, Phylogeny

## Abstract

We formed the Collection of Zoosporic Eufungi at the University of Michigan (CZEUM) in 2018 as a cryopreserved fungal collection consolidating the University of Maine Culture Collection (UMCC, or JEL), the University of Alabama Chytrid Culture Collection (UACCC), and additional zoosporic eufungal accessions. The CZEUM is established as a community resource containing 1045 cryopreserved cultures of *Chytridiomycota*, *Monoblepharidomycota*, and *Blastocladiomycota*, with 52 cultures being ex-type strains. We molecularly characterized 431 cultures by amplifying the majority of the rDNA operon in a single reaction, yielding an average fragment length of 4739 bp. We sequenced multiplexed samples with an Oxford Nanopore Technology MinION device and software, and demonstrate the method is accurate by producing sequences identical to published Sanger sequences. With these data, we generated a phylogeny of 882 zoosporic eufungi strains to produce the most comprehensive phylogeny of these taxa to date. The CZEUM is thus largely characterized by molecular data, which can guide instructors and researchers on future studies of these organisms. Cultures from the CZEUM can be purchased through an online portal.

## Introduction

**Fig. 1:**
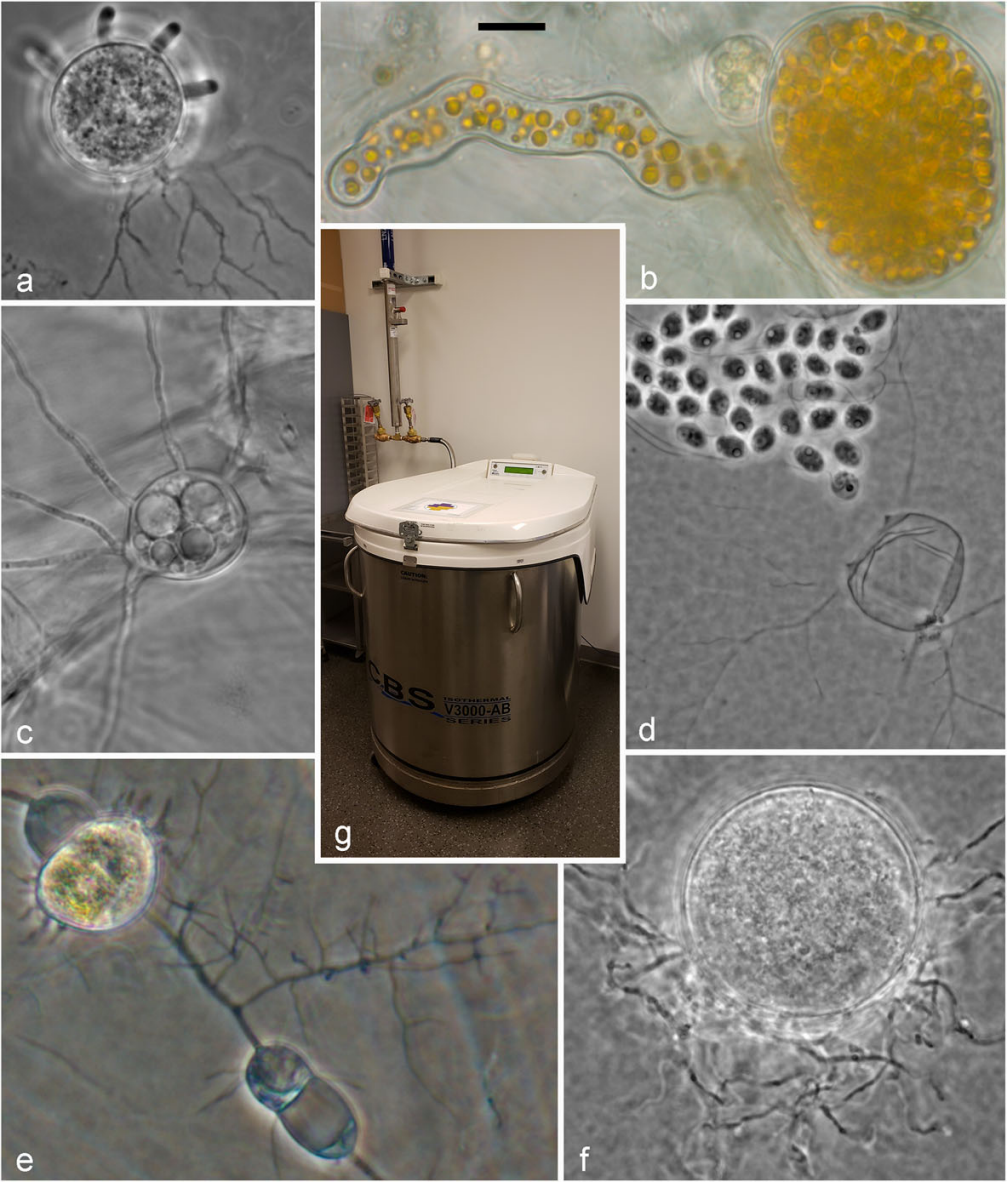
**a** *Gorgonomyces* JEL0965 (*Rhizophydiales*) on agar medium. **b** *Endochytrium* JEL0386 (*Cladochytriales*) on onionskin; measurement bar equals 10 μm for (**a**–**f**). **c** *Rhizophlyctis rosea* JEL0764 (*Rhizophlyctidales*) on onionskin. **d** *Rhizoclosmatium globosum* JEL0006 (*Chytridiales*) releasing zoospores after 24 h on agar medium. **e** *Cladochytrium* JEL0592 (*Cladochytriales*) on soil water medium. **f** *Thoreauomyces humboldtii* JEL0095 (*Spizellomycetales*) on agar medium. **g** Custom BioGenic Systems (CBS) Isothermal V-3000-AB Series cryopreservation freezer containing CZEUM cryopreserved cultures at the University of Michigan RMC

Zoosporic eufungi are members of Kingdom Fungi that reproduce with flagellated spores. The zoosporic fungi form a diverse paraphyletic assemblage at the base of the fungal phylogeny and comprise the superphylum *Chytridiomyceta* (phyla *Chytridiomycota*, *Monoblepharidomycota*, and *Neocallimastigomycota*), and the phyla *Blastocladiomycota*, *Olpidiomycota*, and *Rozellomycota*/*Cryptomycota* (Tedersoo et al. 2018; Naranjo-Ortiz and Gabaldón 2019). Collectively, these microscopic fungi that reproduce with zoospores produced in a sporangium are often referred to as chytrids (Greek for “little pot”). In 2018 the Collection of Zoosporic Eufungi at the University of Michigan (CZEUM) was established with the aim of safeguarding historical culture collections from the two most active research labs in chytrid systematics over the last 30 years. Because these cultures resided in personal collections, the goal was to create a repository with infrastructure that would persist and facilitate continued research on the group. Support from the U. S. National Science Foundation’s program *Collections in Support of Biological Research* made the transition possible. The collection is now housed at the University of Michigan’s Research Museums Complex (RMC) in a modern cryopreservation facility with liquid nitrogen freezers (Fig. 1). Here we describe the contents of the collection from a taxonomic and phylogenetic perspective. Combining existing DNA sequence data with sequencing data we acquired from CZEUM with Oxford Nanopore Technologies (ONT) single-molecule sequencing, we developed a comprehensive phylogeny of a large percentage of isolates in the collection. This growing resource is designed to safeguard a unique collection of significant biodiversity and to support research on zoosporic fungi, a group that is attracting growing interest concerning their ecological roles, cell biology, and pathology, among other topics of research (Longcore and Simmons 2012a, 2020; Powell 2017).

The availability of pure cultures of *Chytridiomyceta* and *Blastocladiomycota* has made possible major advances in our knowledge of these zoosporic eufungi, perhaps the largest being their molecular phylogenetics and taxonomy. The availability of pure cultures facilitated chytrid systematics via transmission electron microscopic studies of zoospores (Barr 1980). More recently, the availability of DNA sequencing data has led to the description of new orders in the last 15 years (Letcher et al. 2006, 2008; Mozley-Standridge et al. 2009; Simmons et al. 2009; Longcore and Simmons 2012b; Karpov et al. 2014, 2016; Seto et al. 2020). Additional orders are likely to exist as evidenced by environmental DNA studies that show a poor match between cultured diversity and uncultured (Freeman et al. 2009; Davis et al. 2016). The proper identification and cataloging of culture collections will not only add to our knowledge of basal fungal diversity, but also will provide DNA information to public databases, information that is necessary to identify and characterize novel diversity detected with environmental DNA sampling techniques.

The most biodiverse collection of zoosporic eufungi was the University of Maine Culture Collection (UMCC, or JEL collection) begun by Joyce E. Longcore in 1984. Longcore was an undergraduate student and employee of Frederick K. Sparrow at the University of Michigan in the 1950s and 1960s, and she began isolating chytrids in the 1980s. At that time, Longcore received training in transmission electron microscopy from Donald J. S. Barr, culminating in the description of new species (Longcore 1992b, 1992a, 1993), one of which, *Podochytrium dentatum* Longcore (1992a), is represented by a viable ex-type culture (JEL0030). After obtaining a Ph.D. from the University of Maine in 1991, Longcore continued culturing and publishing on chytrid systematics (e.g., Longcore 1995; Longcore et al. 1995). In 1997 Longcore was contacted by veterinary pathologists at the Smithsonian National Zoo to help identify a pathogen of frogs; she requested fresh diseased frog skin tissue, from which she isolated the first pure cultures of the amphibian pathogen *Batrachochytrium dendrobatidis* (*Bd*) (Longcore et al. 1999). The availability of *Bd* cultures was pivotal for demonstrating the role of a chytrid fungus in global amphibian declines (Nichols et al. 2001). Longcore’s collaborations over the years increased the UMCC to include *Bd* from multiple locations across the globe. The UMCC was expanded by, and essential to, two NSF PEET projects (DEB-9978094, DEB-0529694). Primarily using cultures housed in the UMCC, Longcore and her student D. Rabern Simmons published revisionary taxonomic treatments of taxa in the *Lobulomycetales* (Simmons et al. 2009, 2012), *Polychytriales* (Longcore and Simmons 2012b), *Spizellomycetales* (Simmons and Longcore 2012), and *Synchytriales* (Longcore et al. 2016).

Contemporaneously, a second chytrid culture collection in the United States was being developed by Martha J. Powell. Powell earned a Ph.D. in 1974 working with William J. Koch at the University of North Carolina. Her work, which focused on zoosporic electron microscopy, was widely recognized and led to her research programs on the systematics and cell biology of chytrids, most recently at the University of Alabama (1997–2019). Powell led the two NSF PEET projects that supported Longcore’s work, and she also received two REVSYS grants (DEB-0516173, DEB-0949305) that allowed the recruitment and training of additional investigators, including longtime collaborator Peter M. Letcher (Ph.D. 2003, University of Alabama Adjunct Associate Professor). Powell maintained chytrid cultures for over 40 years, and established the University of Alabama Chytrid Culture Collection (UACCC), which was widely expanded by Letcher (e.g., PL, PLAUS, and ARG isolates), undergraduate and graduate students, and graduate student William J. Davis (e.g., WJD isolates). The UACCC cultures are integral to over 100 manuscripts on chytrid systematics.

Together, the UMCC and UACCC contain 52 ex-type cultures, represent over 100 years of isolation effort by Longcore, Powell, their colleagues and students, and combined constitute the most biodiverse zoosporic eufungal culture collection in the world, in addition to the largest and most diverse collection of *Batrachochytrium dendrobatidis*. In 2017, Longcore and Powell worked with Simmons and Timothy Y. James to relocate the UMCC and UACCC to the University of Michigan’s Fungarium, a collection within the Herbarium (MICH). With the initial transfer of the UMCC in Jun–Aug of 2018, we established the Collection of Zoosporic Eufungi at the University of Michigan (CZEUM) to ensure the long-term preservation of these collections. The CZEUM grew immediately by the donation of *Allomyces* isolates from the Fungal Genetics Stock Center (FGSC). These cultures, which were collected by Ralph Emerson and Lauritz W. Olson, had been given to the FGSC in 1995 by Lene Lange; 74 isolates from this dried collection are now viably cryopreserved.

One of the goals of the formation of the CZEUM is to fully characterize the diversity of its holdings by sequencing the rDNA operon for each culture. We estimated that 280 JEL strains (excluding *Bd*) and 169 UACCC strains lacked any DNA sequence when accessioned, whereas others lacked either 18S, ITS, or 28S rDNA sequences. In past studies, the amplification of all three of these rDNA regions would necessitate individual amplification reactions and up to twelve sequencing reactions by Sanger sequencing methods. Zoosporic eufungi phylogenetics has been primary based on 28S rDNA sequences, while environmental metabarcoding studies have been primarily conducted with either the more conserved 18S rDNA sequences and species-specific ITS. To provide a unified platform for studying chytrid diversity at many scales, full-length rDNA operon sequencing is required. The Oxford Nanopore Technology (ONT) MinION sequencing device is capable of sequencing long reads of fungal rDNA amplicons generated from a single reaction (Wurzbacher et al. 2018). If replicable across the zoosporic eufungi, amplification of all three regions in a single reaction, followed by nanopore sequencing, would provide a wealth of knowledge with relatively minimal effort and cost, when sequenced in bulk with multiplexed barcoding of pooled amplicons. We have begun to characterize the diversity of the CZEUM with ONT sequencing technology and our results compare favorably with a sample of Sanger sequencing products.

Herein we describe the current holdings of the CZEUM and the efforts made thus far to determine viability and phylogenetic diversity of the cultures therein. We hope to make this fungal collection a leading resource for the study of zoosporic eufungi by providing researchers with vouchered cultures and accepting new cultures for permanent cryopreservation. We have amassed previously-generated GenBank accessions and new ONT sequences from 77% of the publicly available CZEUM cultures (excluding *Bd*), with 61% possessing sequence data for all three (18S, ITS, 28S) rDNA operon regions, and we provide a comparison of ONT sequencing technology versus Sanger sequencing products. Our phylogenetic analysis (Figs. 3, 4, 5, 6, 7, 8, 9, 10, 11, 12 and 13) of the CZEUM holdings plus unavailable cultures is based on newly-generated ONT and published rDNA sequences.

## Methods

### Cryopreservation facility, collection transports, and database construction

The CZEUM is maintained in a cryopreserved state at the University of Michigan RMC cryopreservation unit. The cultures are stored in up to five replicate 1.2 or 2 mL screw-cap cryovials in 81-cell storage boxes in a Custom BioGenic Systems (CBS) Isothermal V-3000-AB Series cryopreservation freezer (hereafter RMC freezer) that is liquid nitrogen cooled (Fig. 1g). At this time, all cultures are maintained in a single freezer; future goals include dividing replicates to other storage units or facilities. No liquid makes contact with the preserved specimens, making their handling relatively easy and safe with minimal personal protective equipment, i.e., cryogloves and face mask. The RMC freezer is on a CBS 2301 Series automated filling system, with daily scheduled fillings, and automated fillings if liquid nitrogen levels reach a set minimum. In the event of low liquid nitrogen levels or high temperatures, RMC staff and appropriate researchers are automatically notified by a CBS Versalert programmed by the cryopreservation unit collection manager. Maximum ultralow temperature is ~ − 190 °C, and should the temperature reach − 150 °C, RMC staff and appropriate researchers are automatically notified via email or cellular data. The cryopreservation unit is on a separate power system so that electricity is maintained when it fails in the building; in a total failure event, RMC staff and appropriate researchers are automatically notified. Without scheduled liquid nitrogen fillings during a power failure, RMC freezers can remain at ultralow temperatures for 14 days, if undisturbed.

We transported the UMCC from the University of Maine overnight to the RMC in Cryoport Express® Dry Shippers (FedEx Deep Frozen Shipping Solution) and transferred cultures to the RMC freezer within 6 h of delivery. We transported the UACCC from the University of Alabama to the RMC overnight on dry ice in a single large cooler containing all cultures and placed cultures in the RMC freezer immediately upon arrival to the RMC. We received *Allomyces* isolates from the FGSC in individual 1.5 mL microcentrifuge tubes containing strips of filter paper, onto which resting spores had been dried.

We transcribed digital and hard-copy notes from the UMCC, UACCC, FGSC, and University of Michigan culture collection into a database in Microsoft Access, which is regularly updated and uploaded to the CZEUM online searchable database (czeum.herb.lsa.umich.edu). The CZEUM collection is integrated into the fungarium of the University of Michigan Herbarium, leveraging shared infrastructure and collection management experience.

### Revitalization and restocking efforts

We prioritized our culture viability check, subsequent growth for restocking, and DNA extraction efforts on (1) cultures in the UMCC for which there was no previously generated molecular data, (2) the UACCC, which had minimal replicates, and (3) the *Allomyces* cultures of the FGSC, this latter group being the focus of additional systematic work beyond the scope of this manuscript. For revitalization of each UMCC and UACCC culture, we removed a single cryovial (1.2 or 2 mL screw-cap vial) from the RMC freezer and placed it on dry ice until thawing at 43 °C for 2 min. If the culture was suspended in liquid medium, we extracted the mixed volume out of the cryovial and placed the sample on two agar plates of the isolate’s preferred medium, as noted by the isolators; we then added an equal volume of sterile distilled water to the agar plate (Boyle et al. 2003). When a polycentric rhizomycelium or Q-tip® (Barr and Babcock 1994) was present in the cryovial, we placed half of the liquid contents of the cryovial on one agar plate and the remaining liquid and fungal/cotton mass from the cryovial on a second agar plate; we then added approximately equal volumes of distilled water to each agar plate. For revitalization of FGSC cultures, *Allomyces* resting spores were stored at − 80 °C until we attempted to revive them by placing a piece of filter paper with resting spores in a well-plate in sterile water. After noting zoospore release, we aspirated the zoospore suspension from the well-plate and inoculated it onto an agar plate of ¼-strength YpSs (Emerson 1958). Once the culture was growing and free of contaminants, the culture was cryopreserved, as below.

**Fig. 2:**
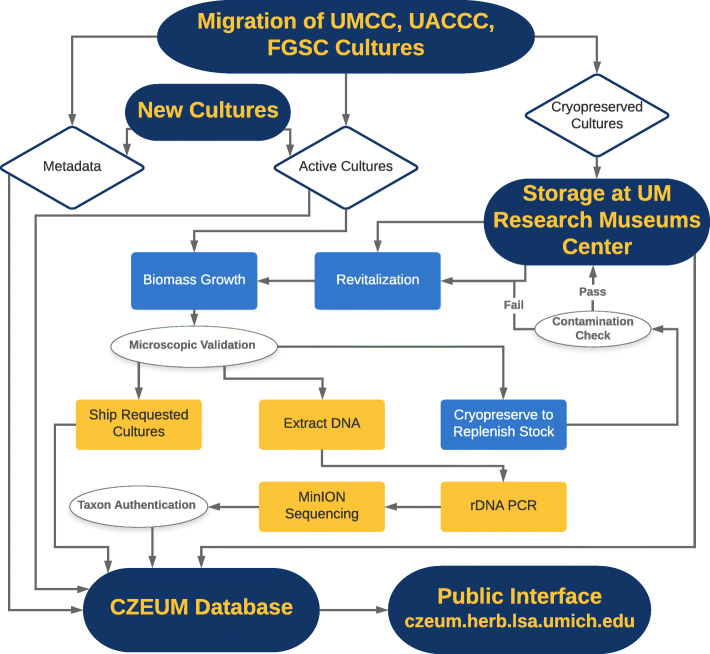
Metadata of collections and new cultures and information on the cultures’ storage (cryopreservation/active status) are kept in the CZEUM database and provided online to the public. Collection maintenance and quality control (blue boxes) and internal and independent research efforts (yellow boxes) are also recorded in the database

Most cultures that were revitalized grew within 24–96 h, but we incubated plates for a minimum of 2 weeks before scoring viability. Isolates that scored as inviable after one revitalization attempt remained in the CBS freezer for repeat processing at a later time. Contaminated cultures were noted and retained for processing at a later time. After we detected growth with a stereoscope (motile zoospores and/or fungal tissue attached to the agar) in revitalized cultures, we assessed the need for additional cryopreserved tissue based on the number of cryovials still available in the RMC freezer. For cultures with fewer than three replicates, we optimally attempted to cryopreserve a total of five replicates, via 10% FBS/DMSO cryopreservation (Boyle et al. 2003) in isopropanol-buffered freezing containers placed at − 80 °C for at least 18 h. We preserved an additional cryovial exceeding our optimal replicate count to allow for a quality control check of our restocking attempt. We thawed this quality control cryovial as described above. If the culture was viable and contaminant-free, the remaining cryovials were placed into the RMC freezer. If the fungus was unviable or contaminated, we made two more attempts to revitalize the tissue before repeating the cryopreservation process with viable, contaminant-free thalli. A workflow of the processes described above is visualized in Fig. 2.

### DNA extraction, amplification, and sequencing

#### DNA extraction

We placed an agar plug of viable culture into ~ 20 mL of each isolate’s preferred sterile liquid medium in 50 mL centrifuge tubes and incubated the culture for 1–2 weeks at room temperature, unless otherwise noted by the isolators. For monocentric taxa, we centrifuged cultures ready for DNA extraction at 4000 RPM at 4 °C for 20 min. After pelleting, we removed most of the supernatant, resuspended the pellet in the remaining < 1.5 mL, and transferred the suspension to a 1.5 mL microcentrifuge tube. We then centrifuged the microcentrifuge tube at 13000 RPM for 5 min and removed the majority of the liquid medium. For polycentric taxa, we removed tissue from the ~ 20 mL of liquid medium with a sterilized probe made of 26 gauge resistance heating wire, placed the tissue directly in the microcentrifuge tube, and centrifuged at 13000 RPM for 5 min before removing the majority of the liquid medium. Once fungal tissue was pelleted and relatively free from liquid medium, we followed a 2X CTAB extraction protocol similar to James et al. (2008), without use of phenol.

#### rDNA amplification

We quantified stock DNA extracts with a Qubit 4 fluorometer (Thermo Fisher Scientific) and diluted working stocks for PCR amplification to 0.25–0.75 ng/μL. To amplify the majority of the 18S-ITS1–5.8S-ITS2-28S rDNA operon, we used the rRNA-PCR primers NS1short/RCA95m described by Wurzbacher et al. (2018), to which we added barcodes to allow multiplexed sequencing (Table S1). We prepared 12.5 μL amplifications with TaKaRa LA Taq® DNA Polymerase kits (Takara Bio USA, Inc.) composed of the following: (1) 2.875 μL UV-radiated PCR water, (2) 1.25 μL 10X LA Taq® buffer, (3) 1.25 μL 25 mM MgCl_2_, (4) 2 μL 10 mM dNTPs, (5) 0.125 μL LA Taq® polymerase, (6) 1.25 μL each barcoded primer NS1short/RCA95m, and (7) 2.5 μL working stock DNA template. We amplified our products on a Eppendorf Mastercycler Pro S with an initial denaturing at 95 °C for 5 min, followed by 35 cycles of denaturing at 95 °C for 30 s, annealing at 55 °C for 30 s, and extension at 68 °C for 4 min, and a final hold at 4 °C. We confirmed amplification of our 4.5–6 kbp products by gel electrophoresis. We pooled 2 μL of successful amplicons in sample sets of 96 amplicons with unique barcode combinations to maximize sequencing efficiency.

#### ONT MinION sequencing

##### DNA library preparation, MinION, and MinKNOW

To sequence each pool of up to 96 barcoded amplicons, we followed the ONT Ligation Sequencing Kit (SQK-LSK109) protocol, briefly described here. We conducted DNA repair and end-prep of our pooled samples with FFPE Repair Mix and Buffer and Ultra II End Prep Mix and Buffer (New England Biolabs® Inc.), followed by purification and size selection by the addition of 60 μL AMPure XP bead solution (Beckman Coulter Inc.). We incubated samples at room temperature for 5 min on a Hula shaker, washed AMPure XP beads twice with freshly-made 70% ethanol after magnetic pelleting, added 60 μL of UV-radiated water and resuspended beads, incubated beads at room temperature for 10 min, magnetic pelleted them for 3 min, and retained 60 μL of the prepared pool in a fresh microcentrifuge tube for immediate ligation or overnight refrigeration. For adapter ligation, we incubated our 60 μL prepared pool with 25 μL ONT ligation buffer, 5 μL ONT adapter mix, and 10 μL T4 ligase from the NEB Quick Ligation Module (New England Biolabs® Inc.) for 45 min at room temperature. We purified and size selected our ligation pool by adding of 40 μL AMPure XP bead solution and incubating at room temperature for 5 min on a Hula shaker. We resuspended beads in two washes of ONT Long Fragment Buffer after magnetic pelleting, removed the supernatant after magnetic pelleting, incubated for 20 min in 16 μL ONT elution buffer, and retained the ligated pool supernatant after magnetic pelleting. We used 1 μL of the ligated pool to quantify the pool on a Qubit 4 fluorometer, and, if necessary, diluted our working ligated pool solution with ONT elution buffer to the recommended optimal molarity (50–100 fmols).

After flow cell priming, we applied our DNA library composed of 37.5 μL ONT sequencing buffer, 25.5 μL ONT loading beads, and 12 μL of our working ligated pool to the flow cell. We conducted the MinION experiments on a Mac OS for 25–310 min (Table S2) in MinKNOW, with live basecalling inactivated. After the experiment concluded, we used an ONT Wash Kit to clean and store the flow cell at 4 °C until its next use.

##### Quality and size filtering, demultiplexing, assembly, and polishing

To get fastq files for downstream processing, we performed basecalling of resulting fast5 files from each MinION experiment in Guppy 3.2.4 (ONT) according to manufacturer-recommended settings for R9.4.1 flow cells. We compiled individual fastq files into a single fastq file for each experiment. We filtered sequences based on length (4–7 kb) and minimum quality score (10) with NanoFilt (De Coster et al. 2018), converted fastq to fasta for downstream processing with Seqtk (https://github.com/lh3/seqtk), and demultiplexed the single experiment fasta file with Minibar (Krehenwinkel et al. 2018). We attempted assemblies of demultiplexed samples in Canu 1.9 (Koren et al. 2017), accepting the output of the first setting that yielded an assembly composed of ≥20 reads, with four settings from the manual and developers’ recommendations (Koren, pers. comm.), listed here by highest to lowest priority: (1) “faster” default setting (overlapper = mhap utgReAlign = true), (2) smash haplotypes setting (corOutCoverage = 200 correctedErrorRate = 0.15), (3) continuity improvement setting (corMhapSensitivity = normal), and (4) metagenomics setting (corOutCoverage = 10,000 corMhapSensitivity = high corMinCoverage = 0). We polished assemblies with Medaka (https://github.com/nanoporetech/medaka) to obtain final rDNA sequences.

#### Sequence comparison to Sanger technology

We initially targeted amplification of UMCC cultures for which there was no previous sequence data; however, we also sequenced taxa with Sanger rDNA sequences in GenBank. To evaluate the use of our ONT sequencing methods, we used the BLAST algorithm to compare archived Sanger data from 136 cultures to our long-read rDNA for the same isolates (Table S3).

### Molecular phylogeny

For our phylogeny of the *Chytridiomyceta* and *Blastocladiomycota*, we focused on phyla held in the CZEUM, i.e. *Chytridiomycota*, *Monoblepharidomycota*, and *Blastocladiomycota*, and thus excluded *Neocallimastigomycota* from the analysis. From GenBank, we selected 28S rDNA sequence data from cultured zoosporic eufungi, and added 18S rDNA sequence data when available from the same culture. This led to rDNA sequence data for 451 cultures (Table S4), to which we aligned ONT rDNA sequences generated from 431 CZEUM cultures (Table S5). We concatenated GenBank sequences in Geneious 9.1.8, aligned the complete data matrix of 882 sequences in MAFFT (v. 7.310), and edited ambiguously aligned regions in TrimAl (v. 1.2). We conducted ML best tree searches and calculated bootstrap percentages from 500 replicates in RAxML (v. 8.2.11). We submitted the TrimAl alignment and best ML with bootstrap support to TreeBASE as Submission 26,377.

## Results

### CZEUM database and collection biodiversity

As of Feb 2020, the CZEUM contains 1045 publicly available cryopreserved cultures, spanning the *Blastocladiomycota* and *Chytridiomyceta* (Table 1). The collection includes 52 species ex-type and lectotype strains and 752 of the cultures have proven viable. The CZEUM can be accessed at czeum.herb.lsa.umich.edu, where it can be searched on multiple fields (e.g., genus, substrate) and strain requests can be made. Strains are distributed for a recharge fee under a fixed, standard material transfer agreement.

**Table 1:** Taxa of available cryopreserved cultures in CZEUM

Phylum	Order	Culture Counts	Genera	Species Ex-type or Ex-Lectotype
*Blastocladiomycota*	*Blastocladiales*	96	*Allomyces*	
			*Blastocladiella*	
			*Catenaria*	
			*Catenomyces*	
			*Catenophlyctis*	
			*Paraphysoderma*	
*Chytridiomycota*	*Chytridiales*	210	*Asterophlyctis*	2
			*Avachytrium*	
			*Chytridium*	
			*Chytriomyces*	
			*Delfinachytrium*	
			*Dendrochytridium*	1
			*Dinochytrium*	
			*Entophlyctis*	1
			*Fayochytriomyces*	1
			*Irineochytrium*	1
			*Obelidium*	
			*Odontochytrium*	1
			*Phlyctochytrium*	
			*Phlyctorhiza*	
			*Physocladia*	
			*Podochytrium*	1
			*Polyphlyctis*	1
			*Pseudorhizidium*	1
			*Rhizoclosmatium*	4
			*Rodmanochytrium*	2
			*Siphonaria*	
			*Wheelerophlyctis*	2
			*Zopfochytrium*	1
	*Cladochytriales*	86	*Catenochytridium*	
			*Cladochytrium*	
			*Cylindrochytridium*	
			*Diplophlyctis*	
			*Endochytrium*	
			*Nephrochytrium*	
			*Nowakowskiella*	
			*Septochytrium*	
	*Lobulomycetales*	10	*Alogomyces*	1
			*Clydaea*	1
			*Lobulomyces*	
	*Polychytriales*	5	*Arkaya*	1
			*Lacustromyces*	
			*Neokarlingia*	
			*Polychytrium*	
	*Rhizophlyctidales*	25	*Borealophlyctis*	
			*Rhizophlyctis*	
			*Sonoraphlyctis*	
	*Rhizophydiales*	436	*Alphamyces*	
			*Angulomyces*	
			*Aquamyces*	
			*Batrachochytrium* (235)^a^	1
			*Betamyces*	1
			*Boothiomyces*	1
			*Coralloidiomyces*	1
			*Gammamyces*	1
			*Globomyces*	1
			*Gorgonomyces*	
			*Homolaphlyctis*	1
			*Kappamyces*	1
			*Operculomyces*	1
			*Paranamyces*	1
			*Protrudomyces*	1
			*Rhizophydium*	1
			*Terramyces*	1
			*Uebelmesseromyces*	
			*Ulkenomyces*	
			*Urceomyces*	1
	*Spizellomycetales*	162	*Brevicalcar*	1
			*Bulbosomyces*	1
			*Fimicolochytrium*	2
			*Gaertneriomyces*	1
			*Gallinipes*	2
			*Geranomyces*	4
			*Kochiomyces*	
			*Powellomyces*	
			*Spizellomyces*	
			*Thoreauomyces*	1
			*Triparticalcar*	1
	*Synchytriales*	3	*Synchytrium*	1
	*Incertae sedis*	1	*Quaeritorhiza*	1
*Monoblepharidomycota*	*Monoblepharidales*	11	*Gonapodya*	
			*Harpochytrium*	
			*Hyaloraphidium*	
			*Monoblepharella*	
			*Telaspaerula*	1
**Totals*:***	**10**	**1045**	**85**	**52**

Ordinal and generic rank based on molecular phylogenetics and isolators’ notes. ^a^indicates culture number of specific genus

### Revitalization, restocking, and DNA extraction

Of the attempted revitalization of 415 UMCC cultures, 369 proved viable (88.92%). Of these, we extracted DNA from 314 UMCC cultures, for which growth was sufficient, and we attempted rDNA PCR amplification from all extracts, resulting in 270 successful amplifications. We attempted ONT sequencing of all 270, and successfully sequenced rDNA for 269 UMCC cultures (99.63%).

Of the attempted revitalization of 341 UACCC cultures, 285 proved viable (83.58%). Of these, we extracted DNA from 177, for which growth was sufficient, and we attempted rDNA PCR amplification of all 177, resulting in 144 successful amplifications. We attempted ONT sequencing of all 144, and successfully sequenced rDNA for 137 cultures (95.14%).

Of the attempted revitalization of 33 *Batrachochytrium* (*Bd*) cultures, mainly from those already previously cryopreserved in the James laboratory at the University of Michigan, 25 proved viable (75.76%). We extracted DNA from 2 *Bd* cultures, and successfully amplified ONT sequences for both cultures. The remaining ONT sequencing efforts were focused on cultures held by the CZEUM, but these cultures (e.g. Barr isolates) are not publicly available due to intellectual property restrictions.

### DNA sequencing and Sanger comparisons

We generated ONT MinION sequences for 431 CZEUM cultures (Table S4). To compare Sanger sequencing products with our ONT sequencing products, we sampled GenBank submissions generated from 136 cultures that are now in the CZEUM (Table S3). The 18S rDNA ONT sequences were 99.79% similar to 64 Sanger sequences, with a range of 97.51–100% similarity. The 28S rDNA ONT sequences were 99.83% similar to 131 Sanger sequences, with a range of 98.33–100% similarity. The ITS rDNA ONT sequences were 99.37% similar to 44 Sanger sequences, with a range of 90.39–100% similarity. Differences between sequences were most commonly observed in single nucleotide repeats, or poorly edited primer sites and unresolved polymorphisms in the Sanger sequence.

### Molecular phylogeny

**Fig. 3:**
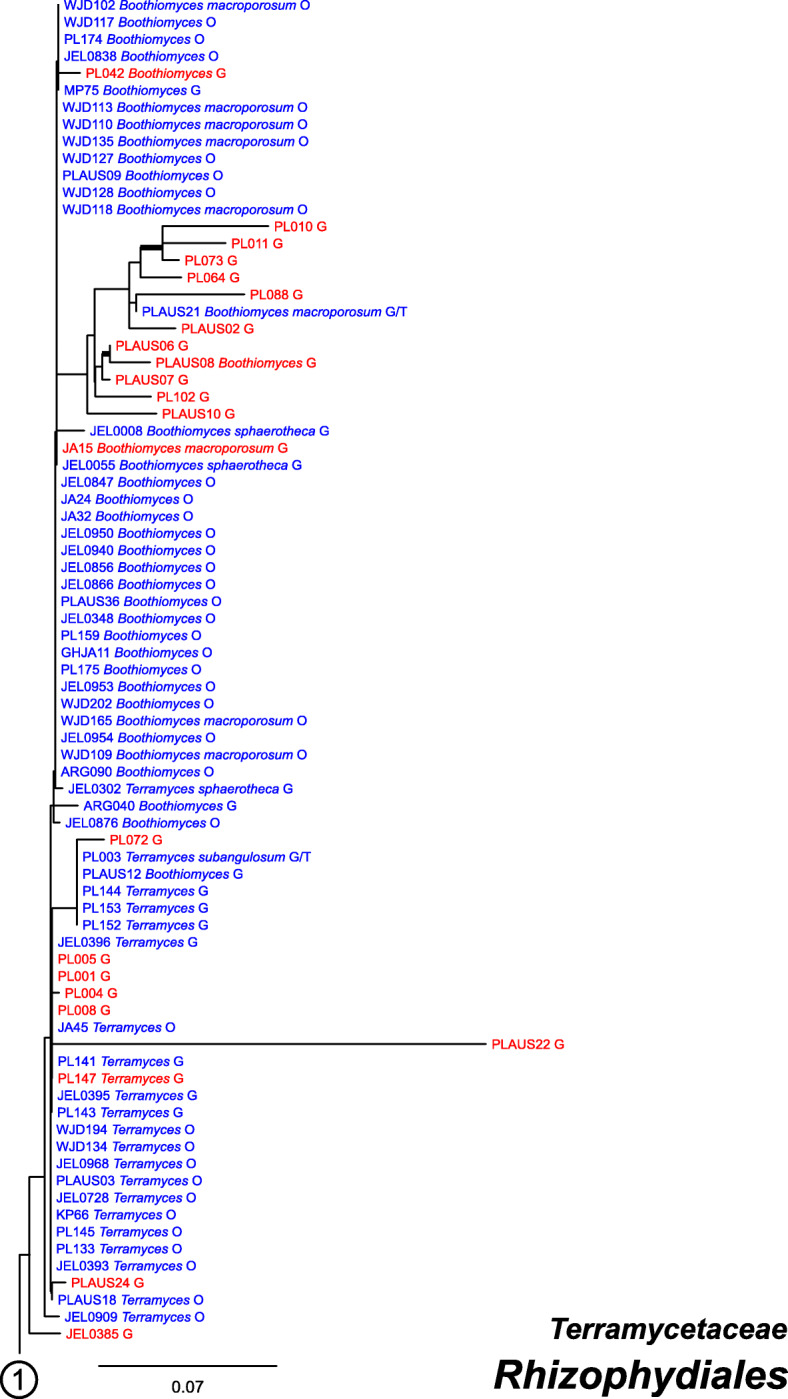
Portion of RAxML phylogeny containing *Rhizophydiales*, family *Terramycetaceae*. For Figs. 3, 4, 5, 6, 7, 8, 9, 10, 11, 12 and 13: Branches with ≥50% ML bootstrap support in bold; cultures available in the CZEUM in blue, unavailable cultures in red; G = published GenBank sequences, O = ONT sequences generated in this study, T = ex-type culture; “//” on branches indicate length reduced by half, “///” reduced to a third of full length, “////” reduced to a quarter of full length

**Fig. 4:**
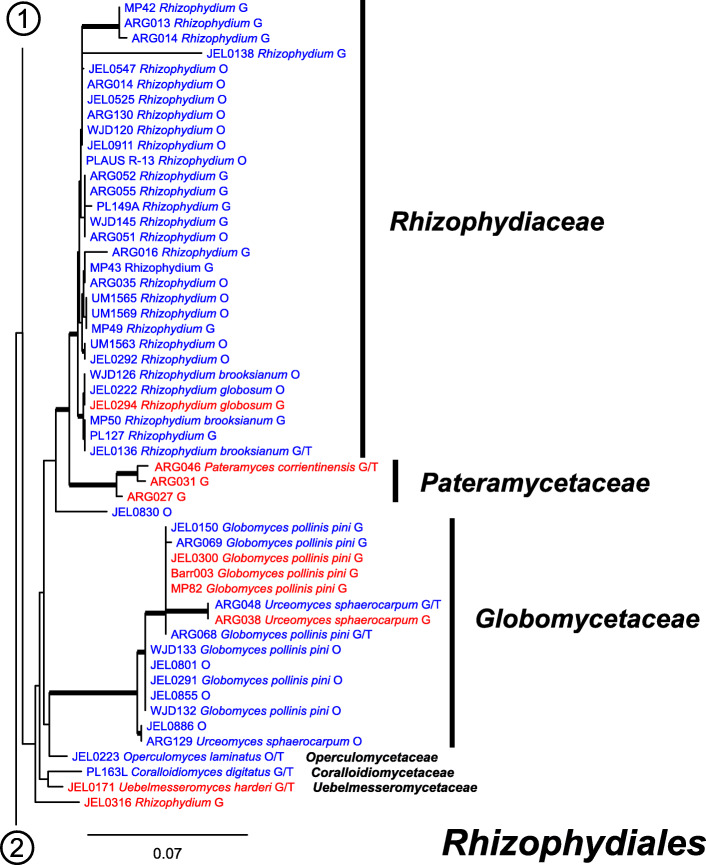
Portion of RAxML phylogeny containing *Rhizophydiales*, families *Rhizophydiaceae*, *Pateramycetaceae*, *Globomycetaceae*, *Operculomycetaceae*, *Corallioidiomycetaceae*, *Uebelmesseromycetaceae*

**Fig. 5:**
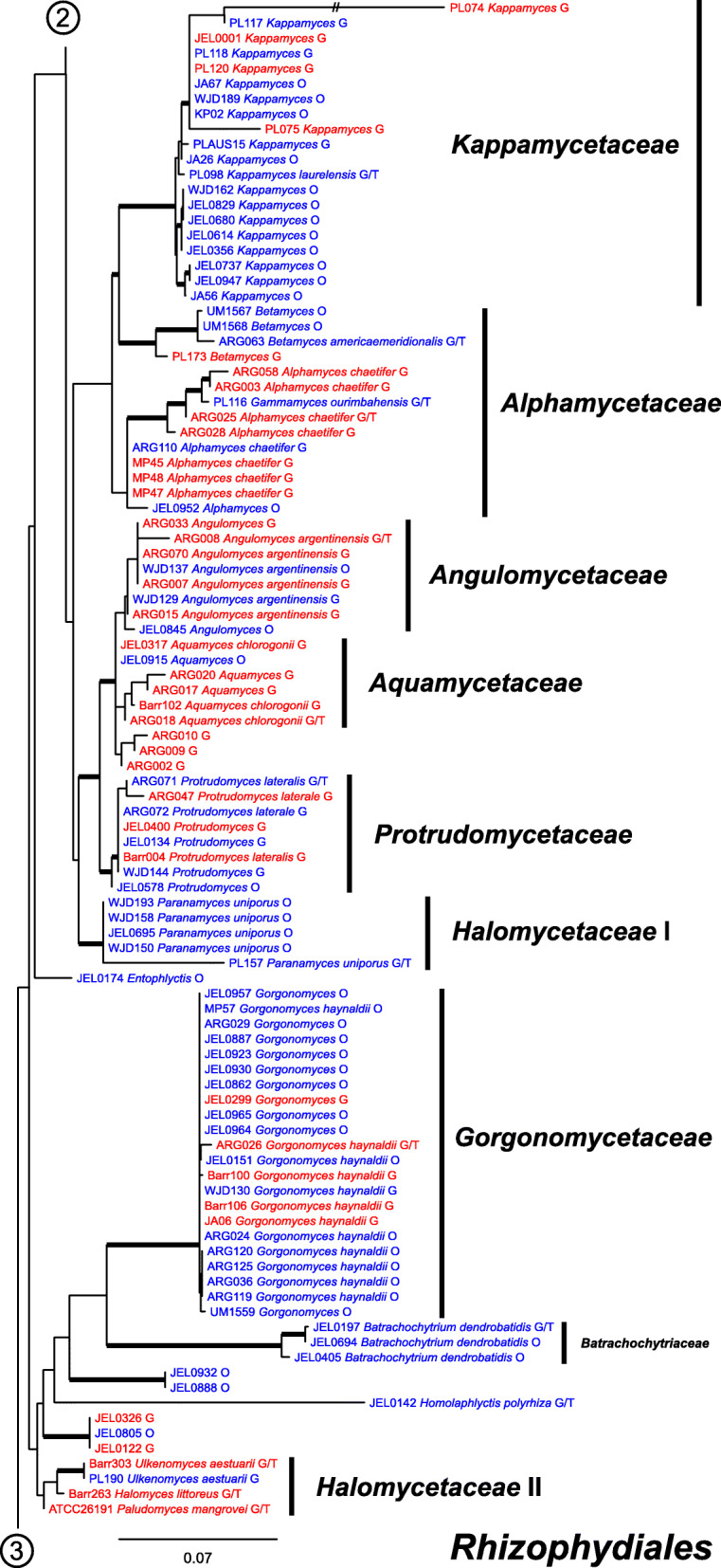
Portion of RAxML phylogeny containing *Rhizophydiales* families *Kappamycetaceae*, *Alphamycetaceae*, *Angulomycetaceae*, *Aquamycetaceae*, *Protrudomycetaceae*, *Halomycetaceae*, *Gorgonomycetaceae*, *Batrachochytriaceae*

**Fig. 6:**
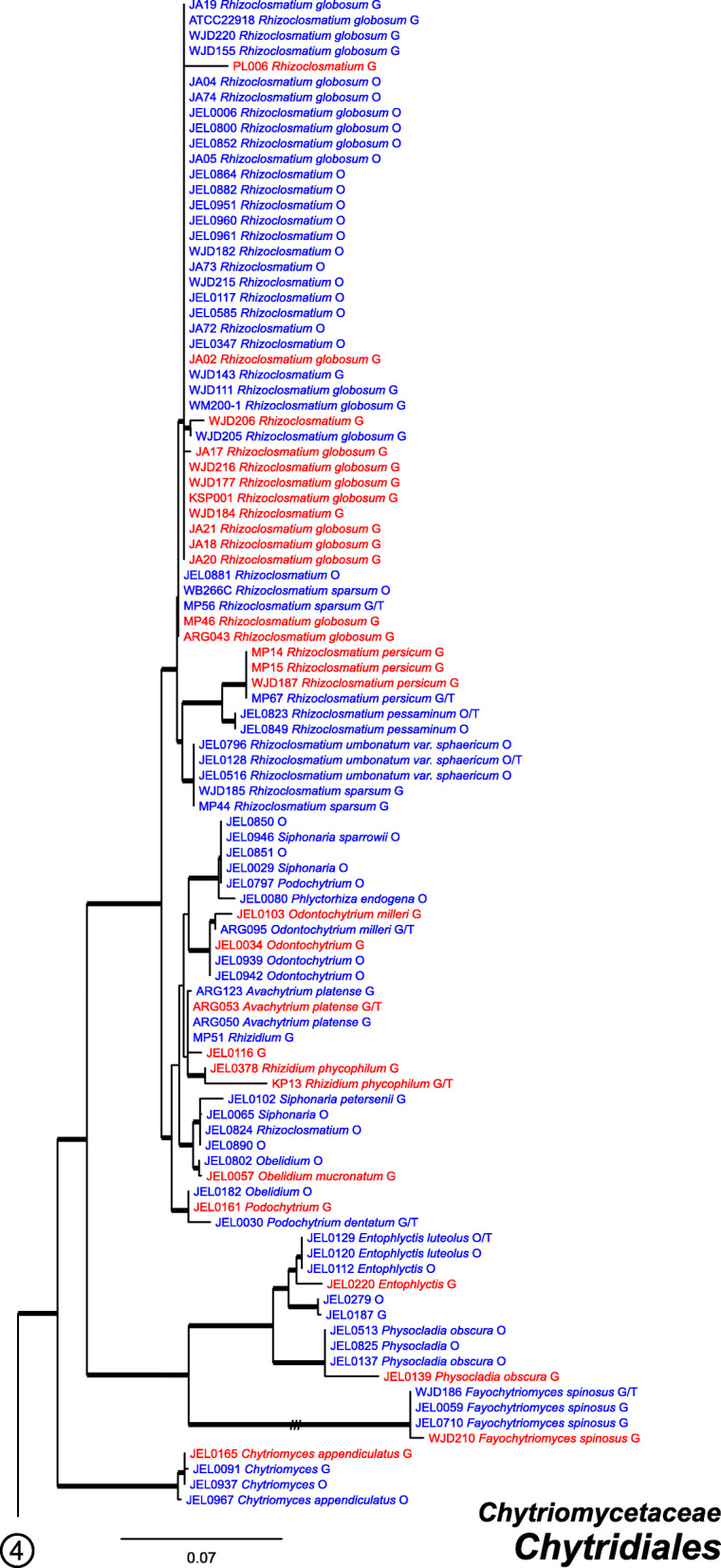
Portion of RAxML phylogeny containing *Chytridiales*, first part of family *Chytriomycetaceae*

**Fig. 7:**
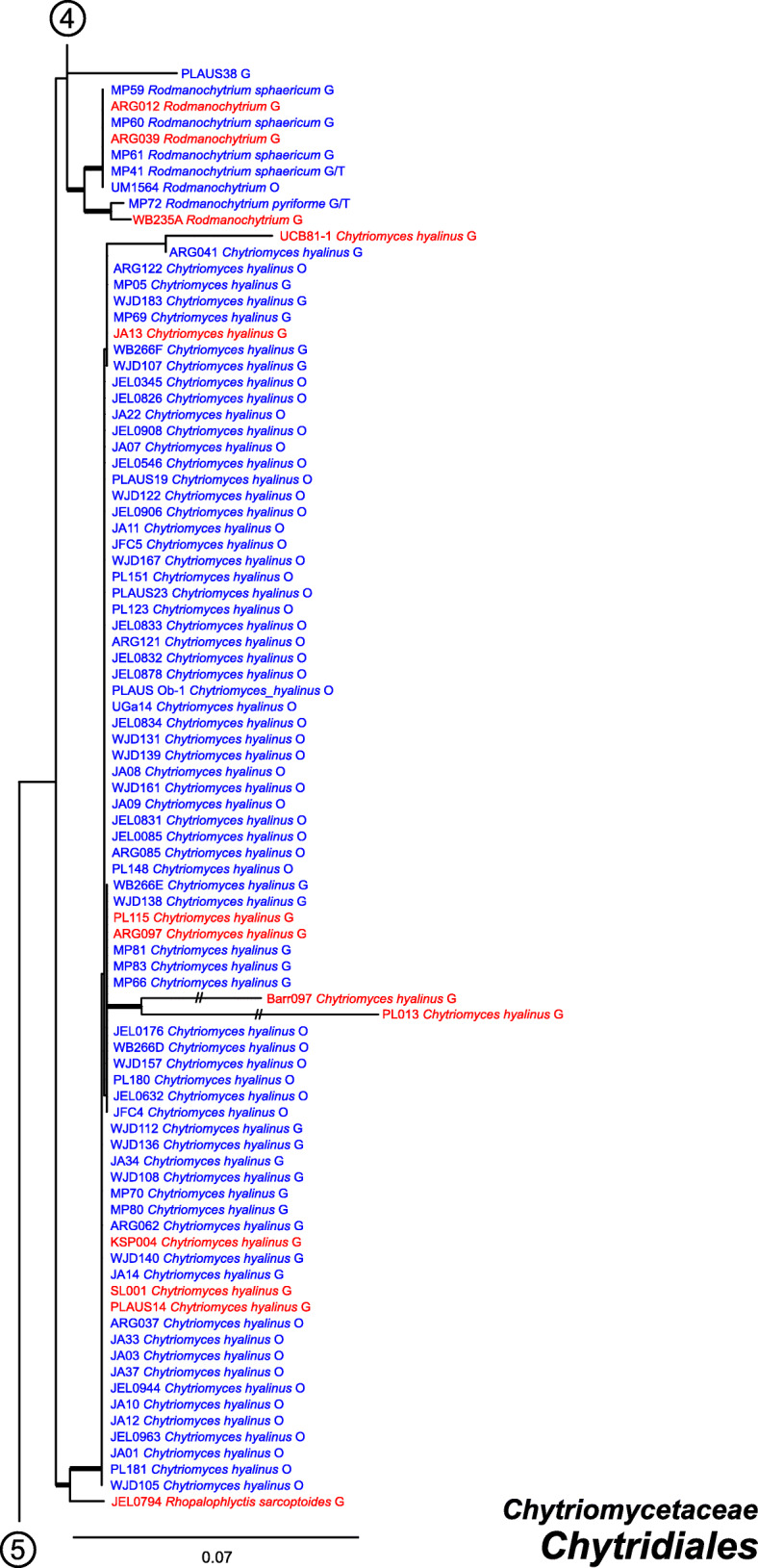
Portion of RAxML phylogeny containing *Chytridiales*, second part of family *Chytriomycetaceae*

**Fig. 8:**
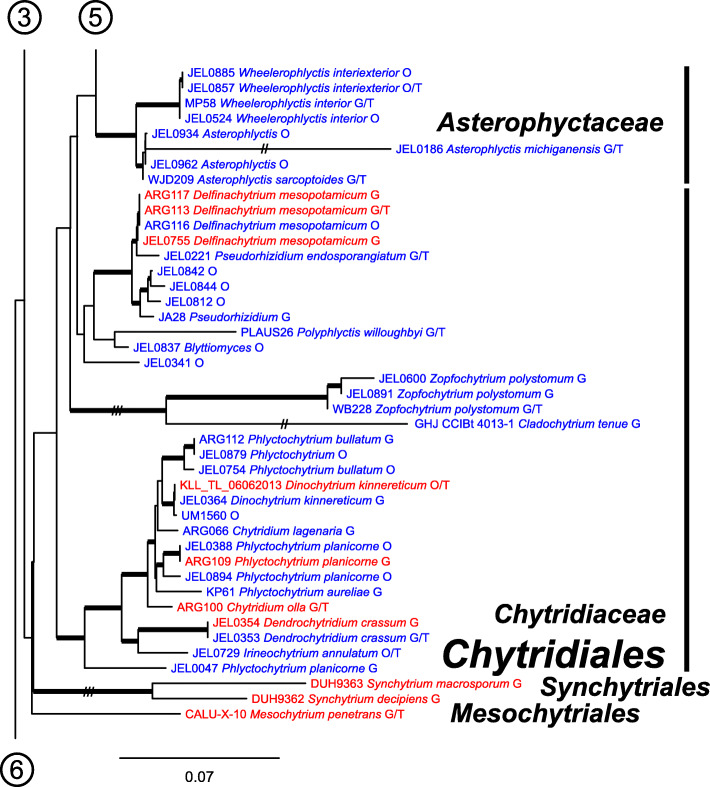
Portion of RAxML phylogeny containing *Chytridiales*, families *Asterophyctaceae*, *Chytridiaceae*, *Synchytriales*, and *Mesochytriales*

**Fig. 9:**
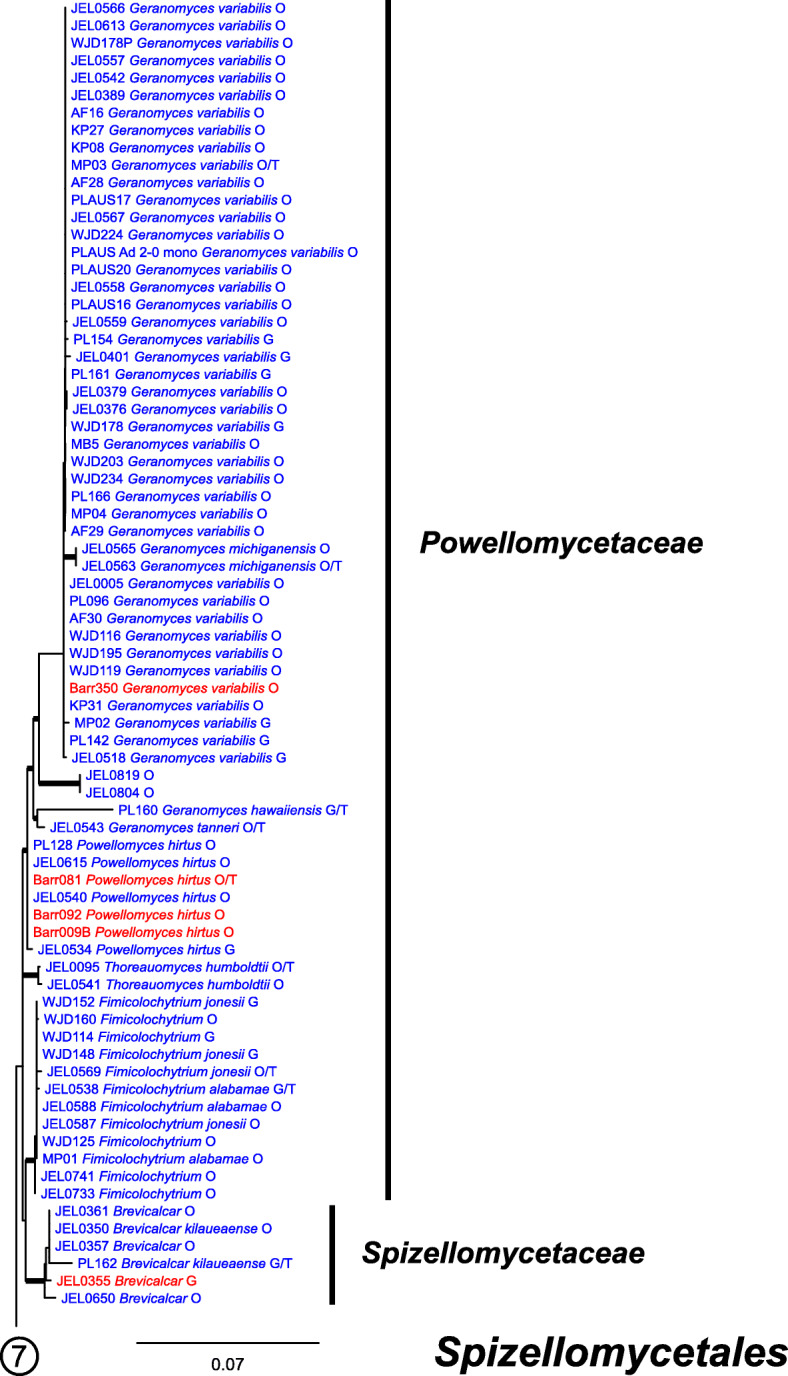
Portion of RAxML phylogeny containing *Spizellomycetales*, family *Powellomycetaceae*, and first part of *Spizellomycetaceae*

**Fig. 10:**
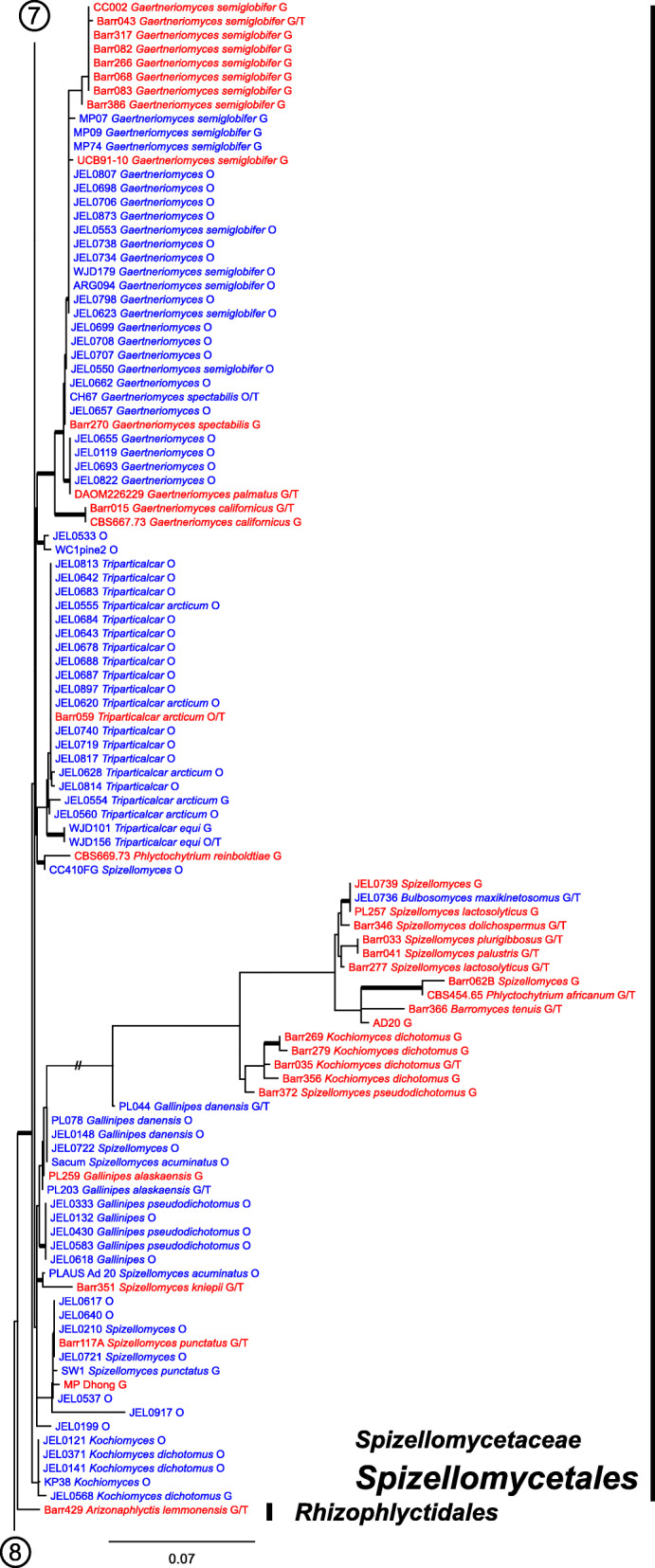
Portion of RAxML phylogeny containing *Spizellomycetales*, second part of *Spizellomycetaceae*, and first part of *Rhizophlyctidales*

**Fig. 11:**
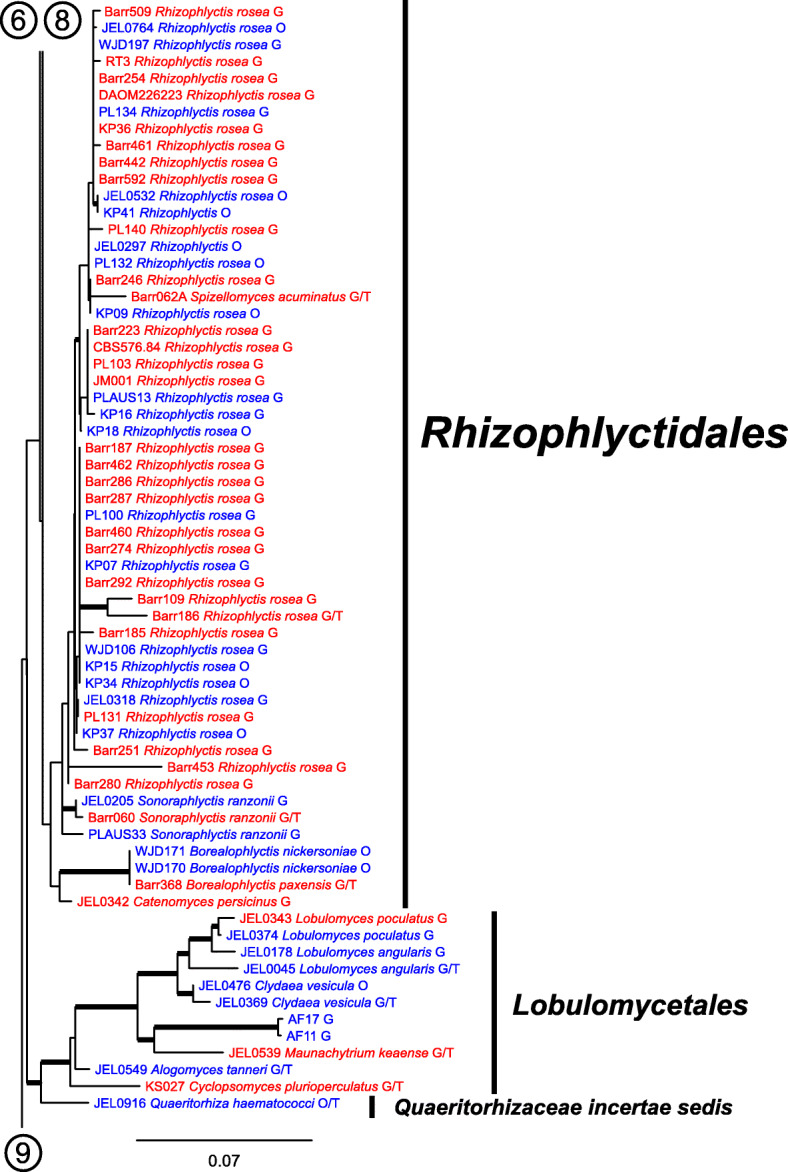
Portion of RAxML phylogeny containing second part of *Rhizophlyctidales*

**Fig. 12:**
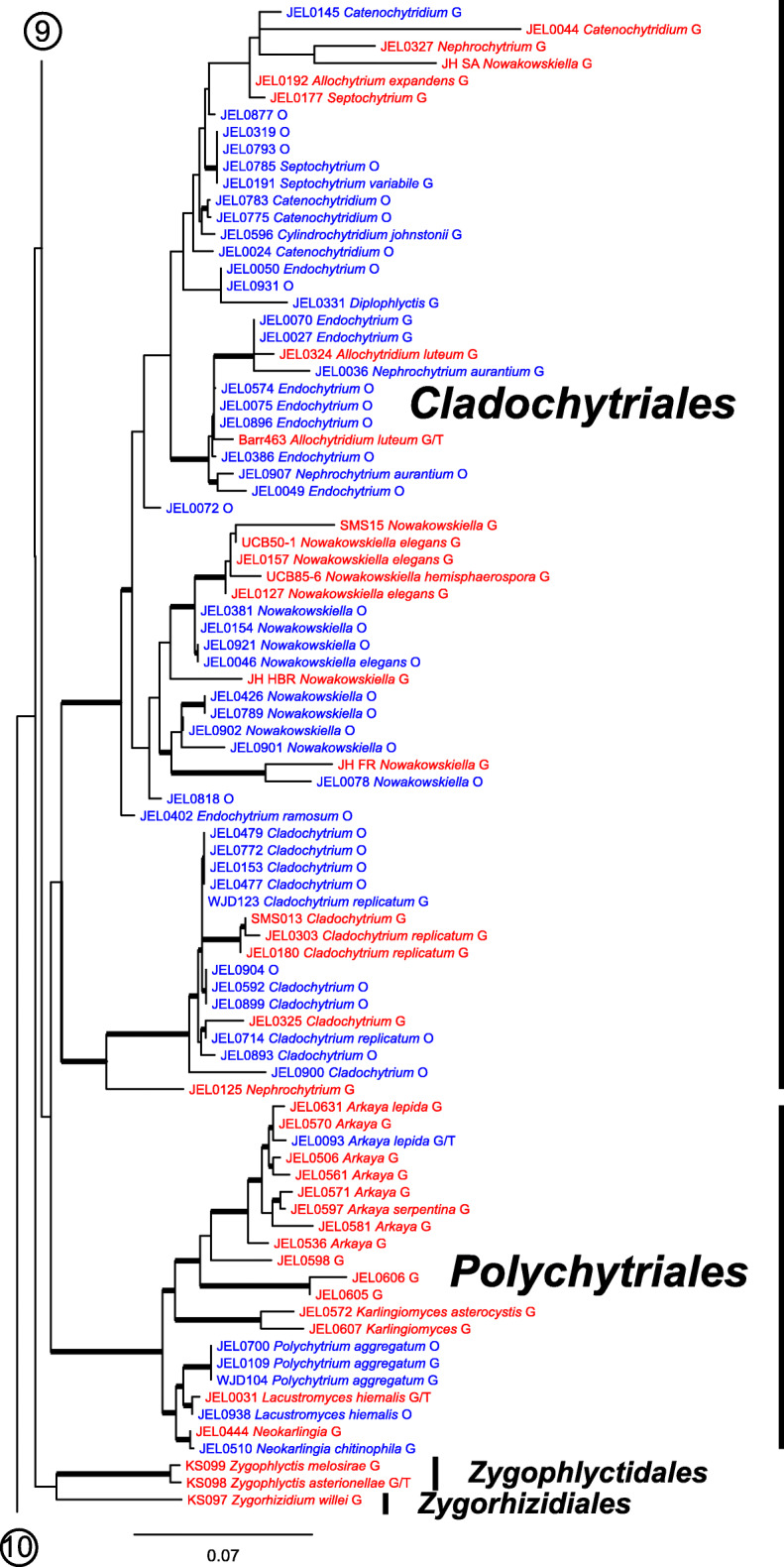
Portion of RAxML phylogeny containing *Cladochytriales*, *Polychytriales*, *Zygophlyctidales*, and *Zygorhizidiales*

**Fig. 13:**
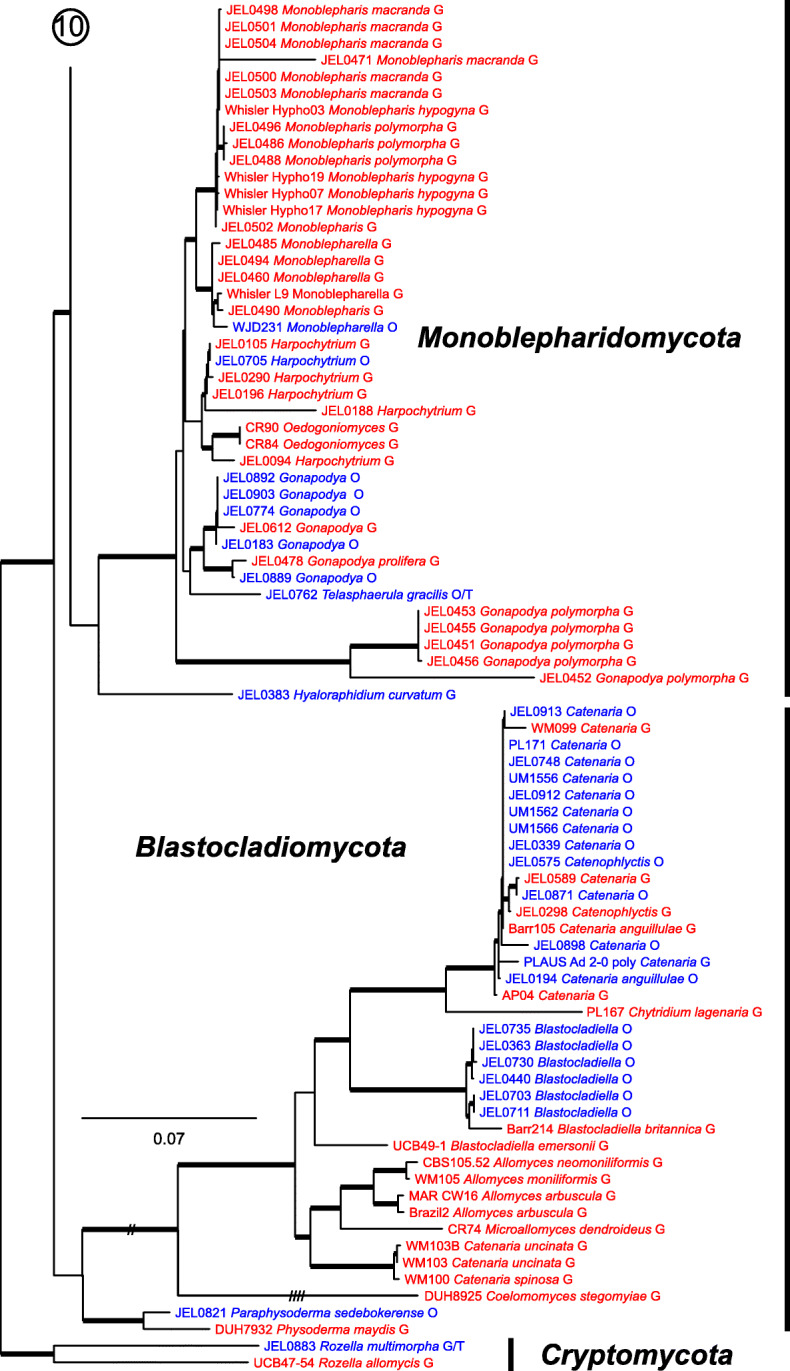
Portion of RAxML phylogeny containing *Monoblepharidomycota*, *Blastocladiomycota*, and *Cryptomycota* (outgroup)

Our ML phylogeny of 882 cultures represents 13 orders of the *Blastocladiomycota* and *Chytridiomyceta*. The *Rhizophydiales* (Figs. 3, 4 and 5) resolve into 15 recognized families and additional lineages. A group consisting of the *Mesochytriales*, *Synchytriales*, and *Chytridiales*, which has three families, (Figs. 6, 7 and 8) is sister to the *Rhizophydiales*. The *Spizellomycetales* (Figs. 9 and 10) resolve into two families and is sister to the *Rhizophlyctidales* (Figs. 10 and 11). The *Lobulomycetales* (Fig. 11) is sister to all orders above, and this group of seven orders is sister to the monophyly of the *Cladochytriales* and *Polychytriales* (Fig. 12), with the *Zygophlyctidales* and *Zygorhizidiales* in a basal lineage of the *Chytridiomycota* (Fig. 12). The *Monoblepharidomycota*, *Blastocladiomycota*, and *Cryptomycota* form distinct clades outside of *Chytridiomycota* (Fig. 13).

## Discussion

### CZEUM database

The CZEUM contains 1045 publicly available cultures, with 752 proven viable. The collection represents at least 85 genera in 10 orders across the *Chytridiomyceta* and *Blastocladiomycota*. The CZEUM database is searchable online (czeum.herb.lsa.umich.edu), and for most cultures it contains information on estimated (by the collections’ previous holders) or documented taxonomic assignments, many of which were putatively determined based on the comprehensive phylogeny (Figs. 3, 4, 5, 6, 7, 8, 9, 10, 11, 12 and 13). Also included for most cultures are recommended growth media and metadata, such as isolator of culture, locality of sample, and ex-type recognition. Further improvements on data collection, such as isolator’s notes, sequence data, and DNA extract availability, will be integrated into the website in the future. Most importantly, the CZEUM website provides instructions for ordering cultures and directions for culturing and cryopreservation of zoosporic eufungal cultures.

### Management and standard operating procedures of the CZEUM

The CZEUM is registered in the World Directory of Collections of Cultures and Microorganisms (WDCM). The CZEUM is managed under the auspices of the University of Michigan Herbarium. Oversight of the collection is provided by the fungal curator with advice from an external advisory board consisting of four scientists. Figure 2 outlines the current practices for handling culture acquisition and requests. We intend to genetically barcode all incoming strains in order to provide confirmation of their placement in the fungal tree. Requests for cultures undergo quality checks and refreshment of cryopreserved stocks after revival and before shipment. We are working on implementing quality standards and management structures following WDCM best practices.

### ONT MinION sequencing

On average across eight experiments (Table S2), the MinION took 2.05 min to generate 3843 reads per amplicon. Though this seems to be a high output, quality filtering and assembly steps eliminated many reads from consideration, rarely assembling final products from more than 100 reads. Our low success rate in later experiments also could be correlated to the life span of one flow cell, which was used for experiments CZEUM04 through CZEUM08. During the course of this and other projects from 2017 to 2019, we purchased the ONT MinION, 3 flow cells, two ligation kits, and two wash kits for $2748 USD. In total, we successfully sequenced rDNA sequences for 534 cultures or multiple displacement amplification samples, over 2.5 Mb nucleotides, with an average fragment size of 4739 bp. This translates to a cost of $0.001086 USD per nucleotide, averaging $5.15 USD per sequence and $1.72 USD per region (i.e., ITS, 28S, 18S). Costs will certainly reduce over time, making ONT an attractive option for future, rapid, and accurate sequencing.

Our rDNA sequences generated by ONT compared favorably to sequences generated by Sanger sequencing (Table S3). Our 18S and 28S rDNA sequences were ~ 99.8% similar to published sequences on average, generally indicating one or two insertions or polymorphisms between the two sequences. Errors were commonly associated with differences in single-nucleotide repeats, or poorly edited primer sites and unresolved polymorphisms in the Sanger sequence. Our ITS rDNA sequences were less similar, (99.37%). We believe that this may reflect the increased substitution rate and heterogeneity in the ITS1 and ITS2 regions. The two cultures with the lowest ITS rDNA similarities (ARG029: 95.07%; JEL0333: 90.39%) have other Sanger rDNA sequences with over 99% similarity to our ONT sequences, and removal of these outliers increases the average similarity of the ITS rDNA sequences to 99.69%. For large-scale phylogenetic analyses, these errors are within a range of acceptability and should have little effect on interspecific relationships, and we believe that ONT sequencing is a suitable alternative to Sanger sequencing.

### A phylogenetic tour of the CZEUM collection

#### Comprehensive phylogeny

Our phylogeny (Figs. 3, 4, 5, 6, 7, 8, 9, 10, 11, 12 and 13) of rDNA sequences from 882 cultures constitutes the largest phylogeny of the *Chytridiomyceta*. The *Blastocladiomycota* and *Chytridiomyceta* resolve into 13 recognized orders, and we discuss taxa of particular interest below. The *Rhizophydiales* (Figs. 3, 4 and 5), *Chytridiales* (Figs. 6, 7 and 8), *Spizellomycetales* (Figs. 9 and 10), and *Cladochytriales* (Fig. 12) make up the bulk of the phylogeny and the CZEUM (Table 1). Cultures are also available for *Polychytriales*, *Rhizophlyctidales*, *Lobulomycetales*, *Synchytriales* (*Synchytrium microbalum* cultures not shown in phylogeny, see below), as well as *Blastocladiales*. Well-supported branches for ordinal divisions are lacking, suggesting phylogenomic approaches may be needed, but our phylogeny can serve as a guide in taxon selection, and cultures can be provided by the CZEUM for research and mycological instruction.

#### Rhizophydiales

The *Rhizophydiales* (Figs. 3, 4 and 5) contains 15 families in our phylogeny, though *Pateramycetaceae* is not present in the CZEUM. Some of the families are not monophyletic; for example, *Halomycetaceae* (Fig. 5) is not monophyletic in our phylogeny, which underscores the lack of support for the monophyly of the family in the original description (Letcher et al. 2015). The CZEUM includes a large number of *Batrachochytrium dendrobatidis* (*Bd*) cultures (Table 1), and although most have yet to be tested for viability, the species has a high revival rate from liquid nitrogen storage. Some lineages in our phylogeny need taxonomic attention. For example, JEL0888 and JEL0932, sister to the *Gorgonomycetaceae*-*Batrachochytriaceae* clade, have polycentric growth, the first known examples of that growth pattern in the *Rhizophydiales*. Taxonomic attention to this newly-recognized chytrid fungus is forthcoming.

#### Chytridiales

The *Chytridiales* (Figs. 6, 7 and 8) contains three families, all of which are represented in the CZEUM. The collection has a large number of *Chytriomyces hyalinus* cultures (Fig. 7), indicative of its ubiquity in nature and its relative ease to culture, making this species of interest for studies on population genetics, particularly as it is believed to have a sexual cycle observable in the lab (Moore and Miller 1973). The phylogeny suggests there may be undescribed genera and species in the collection, e.g. JEL0279 and JEL0187 perhaps constitute a new species of *Entophlyctis* in the *Chytriomycetaceae* (Fig. 6), and, under more rigorous examination, JEL0341 in the *Chytridiaceae* may constitute a new genus (Fig. 8).

#### Synchytriales

The *Synchytriales* (Fig. 8) is here represented by two parasitic *Synchytrium* species, neither held within the CZEUM. Longcore et al. (2016) described *S. microbalum*, isolated from pollen, as the first-known non-parasitic species of the order. The CZEUM holds three viable cultures of *S. microbalum*, including the ex-type strain, which is only represented by an 18S rDNA sequence on GenBank. Our attempts at long-read amplifications of these cultures using primers described by Wurzbacher et al. (2018) failed, and thus these cultures failed to meet the criteria for inclusion in our phylogeny. Though this observation is anecdotal, this failure may illustrate that these primers, like most, are not universal across the *Chytridiomycota*. Most published Sanger 28S rDNA sequences do not extend to the RCA95m primer binding site used in our long-read reactions, so modification of the RCA95m primer to broaden its utility across the *Chytridiomycota* will require generation of additional 28S data from other strategies.

#### Spizellomycetales

The *Spizellomycetales* (Figs. 9 and 10) is comprised of the *Powellomycetaceae* and *Spizellomycetaceae*, two families divided by exogenous and endogenous growth. Practically all genera in this order have been delineated on ultrastructural characters, studies that relied heavily upon cultured isolates with easily obtained zoospores. Similar to *Chytriomyces hyalinus* in the *Chytridiales*, *Geranomyces* is a widely distributed, easily cultured chytrid that could be utilized in population genetics studies. *Spizellomycetales* cultures are generally robust and can release zoospores within 24 h after revitalization from cryopreservation. In addition to their research potential, cultures of *Spizellomycetales* are excellent for mycology instructors, who have limited time and resources to devote to maintaining more difficult chytrid fungal cultures for classroom use.

#### Quaeritorhiza

*Quaeritorhiza haematococci* (Fig. 11) is a recently described parasite of *Haematococcus pluvialis* (Longcore et al. 2020). This genus and its family, *Quaeritorhizaceae*, are *incertae sedis* (Table 1) due to lack of support of its placement in a 18S + 28S rDNA phylogeny. However, *Q. haematococci* is supported as sister to the *Lobulomycetales* in our phylogeny. Based on zoosporic ultrastructure, *Q. haematococci* is not likely to be considered a member of the *Lobulomycetales* and may be elevated to ordinal status after further phylogenetic investigation.

#### Cladochytriales

Despite the recognition of their genera and species for longer than many other taxa, the *Cladochytriales* and *Polychytriales* (Fig. 12), both of which contain both monocentric and polycentric species, are among the most understudied orders of the *Chytridiomycota*. Unfortunately, many *Polychytriales* species grow poorly on agar media, and most do not survive cryopreservation attempts. The *Cladochytriales*, however, are more resilient to cryopreservation, and they are the fifth largest constituents of the CZEUM (Table 1). Families within the *Cladochytriales* have been in need of taxonomic revision since the ordinal description by Mozley-Standridge et al. (2009). To begin that process, we have more than doubled the representation of the order through our ONT sequencing. As in other widely sampled orders, our phylogeny also brings to light undescribed lineages in the *Cladochytriales*, e.g., JEL0072, JEL0818, that are likely candidates for additional genera.

## Conclusions

The CZEUM is the largest culture collection of the *Chytridiomycota*, *Monoblepharidomycota*, and *Blastocladiomycota* in the world. With the collaboration of their initial isolators, we have consolidated these cultures at a centralized and safeguarded location at the University of Michigan RMC. We have begun to molecularly characterize this collection with efficient ONT sequencing methods to determine the true biodiversity of the collection. Accession of new cultures into the CZEUM is free of charge, and we have already received cultures for inclusion in the CZEUM from national and international researchers. The phylogenetic diversity of the CZEUM covers most of the known chytrid diversity, yet only a fraction of the strains has been explored for their biological properties. It is our hope that the CZEUM will be used for a broad range of applications, including investigations into cell biology, alpha-taxonomy, physiology, phylogeny, and amphibian disease. Some isolates are appropriate for demonstrating zoospore motility and other morphological characteristics, and we expect to develop a set of cultures that can be ordered to introduce students to the *Chytridiomyceta* and *Blastocladiomycota* in introductory and advanced mycology courses.

## Supplementary information

**Additional file 1: Table S1.** Barcode indexes adapted to Wurzbacher et al. 2018 primers NS1short/RCA95m for sample multiplexing.

**Additional file 2: Table S2.** ONT sequencing attempts, output, and sequencing success after assembly and polishing. Experiments included re-attempts of previously-failed sequencing attempts, along with sequencing attempts not relevant to this project, resulting in a higher total of sequences than reported in this manuscript.

**Additional file 3: Table S3.** Comparison of ONT rDNA sequences to published Sanger rDNA sequences of CZEUM cultures.

**Additional file 4: Table S4.** Published Sanger rDNA sequences used for *Blastocladiomycota* and *Chytridiomyceta* phylogeny.

**Additional file 5: Table S5.** ONT rDNA sequences used for *Blastocladiomycota* and *Chytridiomycota* phylogeny.
